# Unregulated Substance Abuse and Systemic Inflammation Markers: A Review

**DOI:** 10.3390/healthcare14020232

**Published:** 2026-01-16

**Authors:** Carmen Lara-Apolinario, Jose Barroso, Jose Carlos Rodríguez-Gallego, Pedro C. Lara

**Affiliations:** 1 Medicine Department, Fernando Pessoa Canarias University, 35450 Guía, Spainjbarroso@ufpcanarias.es (J.B.);; 2 Preventive Medicine and Public Health Department, Infanta Elena University Hospital, 28342 Madrid, Spain; 3 Psychology Department, Fernando Pessoa Canarias University, 35450 Guía, Spain; 4 Immunology Department, Dr Negrín University Hospital, 35010 Las Palmas de Gran Canaria, Spain; 5 Canarian Comprehensive Cancer Center, San Roque University Hospital, 35010 Las Palmas de Gran Canaria, Spain; 6 Canarian Institute of Cancer Research, 38320 La Laguna, Spain

**Keywords:** NLR, PLR, systemic inflammation, unregulated substance, abuse

## Abstract

**Aim:** There is an urgent need for systematic and well-designed studies to clarify the role of systemic inflammatory parameters, especially the neutrophil–lymphocyte-ratio (NLR), in the pathophysiology and clinical management of unregulated substance addiction. This review aims to synthesize current evidence on the relationship between unregulated substance addiction and systemic inflammatory parameters, focusing specifically on the NLR as a potential biomarker. **Methods:** To ensure a transparent approach in the collection of evidence, this review was carried out following the recommendations of the PRISMA 2020 guidelines and registered in PROSPERO (CRD420251151136). We searched the PubMed and Scopus databases in July2025 using combinations of MeSH terms and keywords related to unregulated substance use and inflammatory biomarkers. The strategy included terms such as “cocaine,” “cannabis,” “opioids,” “heroin,” “fentanyl,” “methadone,” “buprenorphine” “nitazene”, “MDMA”, and “methamphetamine,” combined with “neutrophil-to-lymphocyte ratio.” Filters were applied to limit results to human studies published between 2015 and 2025 in English. The methodological quality of the studies included was assessed using the STROBE 22-item checklist. **Results:** Fifteen studies were included in this review. Methamphetamine and opioid users showed higher NLR and MLR values. For cocaine abuse, although the evidence is limited to a single population-based study, a significant increase in NLR was reported. Controversial results were observed for cannabis use. **Conclusions:** Systemic inflammation markers are related to unregulated substance abuse disorders; however, the sparse available evidence encourages the need for well-designed large, prospective clinical trials.

## 1. Introduction

Substance use disorders represent a significant burden on global health, characterized by compulsive seeking behaviors and substance use despite adverse consequences [1]. These disorders are intrinsically linked to pathological alterations in brain regions that govern reward, motivation, memory, and decision-making, leading to persistent behavioral changes [1]. Addiction is recognized as a chronic and recurrent brain disease, distinguished by an intense desire for substances and, in severe cases, participation in risky behaviors that can result in high rates of relapse even after prolonged periods of abstinence [2]. This complex pathology is driven by a profound dysregulation of motivational circuits, including alterations in the dopamine and opioid peptide systems within the basal ganglia during the binge/intoxication stage, and the activation of stress neurotransmitters in the extended amygdala during withdrawal [3].

In addition, the profound impact of addiction extends beyond neural circuits, encompassing systemic physiological changes, notably chronic inflammation, which has emerged as a crucial factor in both the initiation and perpetuation of substance dependence [4]. This systemic inflammation, characterized by elevated inflammatory markers, is increasingly recognized as a key modulator of addiction pathology, influencing neuroinflammation, neuronal plasticity, and treatment outcomes [5].

The consumption of unregulated substances is a relevant risk factor for the activation of systemic and central inflammatory processes, with implications for the pathophysiology and prognosis of addictive disorders. At the systemic level, chronic unregulated substance use is associated with alterations in inflammatory biomarkers, including C-reactive protein (CRP), IL-6, TNF-α, and hematological parameters such as neutrophil/lymphocyte ratio (NLR), systemic immune inflammation index (IBS), and white blood cell count [6,7,8].

From a biological perspective, various psychoactive substances (cocaine, opiates, cannabis, amphetamines) induce immune cell activation in both the central nervous system and the periphery, highlighting the role of microglia and the modulation of pathways such as the NLRP3 inflammasome, TLR/NF-κB, and the production of proinflammatory cytokines (IL-6, TNF-α, IL-10) [9,10,11,12,13]. The xenobiotic hypothesis posits that these substances are recognized as external agents, triggering protective immune responses that can perpetuate neuroinflammation and disrupt neuronal homeostasis [12,13].

On the other hand, prolactin (PRL) has been studied as a possible immunomodulatory biomarker in addictions, although its specific role in systemic inflammation requires further research [7]. Aberrant expression of microRNAs (miR-155, miR-187) and modulation of cytokines in chronic opioid users reinforce the idea of complex, dose-dependent immunomodulation [14].

Taken together, current evidence suggests that systemic inflammation induced by unregulated substance results from interactions between central and peripheral immune mechanisms, with detectable alterations in hematological and molecular biomarkers that may contribute to the progression and severity of addictive disorders [6,7,8,9,10,11,12,13,14,15].

Research on the relationship between unregulated substance addiction and systemic inflammatory parameters, in particular the neutrophil/lymphocyte ratio (NLR), represents an emerging area of biomedical interest. While there is consensus that substance use disorders are associated with immunological alterations and a proinflammatory state [6,7,8,9,10,11,12,13,14], the available evidence is limited and heterogeneous, especially regarding simple hematologic biomarkers such as NLR [5,6,7,8,9,10,11,14]. Current literature shows that patients with addictive disorders have elevations in proinflammatory cytokines and other peripheral markers; however, results are inconsistent between different substances and study populations [5,16].

Regarding cannabis abuse, a recent meta-analysis found no significant differences in NLR between users and controls, although alterations in other hematological parameters were observed, suggesting a complex modulation of the immune system [17]. Concerning psychostimulants and opioids, data on NLR and other inflammatory biomarkers are scarce and have high methodological variability [15,18,19].

In addition, the lack of longitudinal studies and the absence of consensus on the clinical utility of these biomarkers make their application in clinical practice and translational research difficult [7,15,18,19]. The identification of reliable biomarkers, such as NLR, could facilitate diagnosis, risk stratification, and therapeutic follow-up in patients with addiction, but there is currently insufficient information to establish solid recommendations [7,15,18,19].

Therefore, there is an urgent need for well-designed studies to clarify the role of systemic inflammatory parameters, especially NLR, in the pathophysiology and clinical management of unregulated substance addiction. This review seeks to address this knowledge gap and contribute to the consolidation of evidence that will allow progress towards a more personalized medicine based on biomarkers in the field of addictions [7,15,18,19].

This review aims to synthesize current evidence on the relationship between unregulated substance addiction and systemic inflammatory parameters, focusing specifically on the neutrophil–lymphocyte (NLR) ratio as a potential biomarker.

## 2. Methods

To ensure a transparent approach to the collection of evidence, this review was carried out following the recommendations of the PRISMA 2020 guideline [20] and registered in PROSPERO (CRD420251151136).

### 2.1. PECO Research Question

First, the research question was defined using the PECO approach (Table 1), with the aim of exploring whether the use of unregulated substances is associated with an increase in markers of systemic inflammation (Table 2), especially the NLR, compared to non-users.

### 2.2. Search Strategy

We searched the PubMed and Scopus databases in September 2025 using combinations of MeSH terms and keywords related to unregulated substance abuse and inflammatory biomarkers. The strategy included terms such as “cocaine,” “cannabis,” “opioids,” “heroin,” “fentanyl,” “methadone,” “buprenorphine” “nitazene” “MDMA”, and “methamphetamine,” combined with “neutrophil-to-lymphocyte ratio.” Filters were applied to limit results to human studies published between 2015 and 2025 in English.

Detailed search strategies can be found in Supplementary Material Table S1. In addition, we checked the references of included studies to identify potentially eligible articles.

### 2.3. Eligibility Criteria and Selection of Studies

We incorporate observational research examining the impact of unregulated substance abuse on proinflammatory markers. Inclusion criteria are described in Table 1.

In addition, we excluded studies based on these criteria (1): Studies that included hematological parameters but did not quantify variations in proinflammatory indicators between subjects and control groups. (2) Replication of studies or sharing of participant data. (3) Studies classified as reviews, editorials, papers, case series/reports, secondary analyses, or animal experiments. (4) Studies that used qualitative research methodologies.

Study selection was carried out in two phases: screening of titles and abstracts, followed by full-text evaluation. This process was carried out independently by two reviewers (Lara-Apolinario C and Lara PC), with discrepancies resolved by consensus.

### 2.4. Data Extraction

For data extraction, a standardized spreadsheet was used to record information on study design, sample size, population characteristics, comparators, biomarkers evaluated, and main results. Given the heterogeneity of designs and outcomes, a narrative synthesis was chosen following the recommendations of PRISMA 2020.

### 2.5. Quality

The methodological quality of the included studies was assessed using the STROBE 22-item checklist, recommended for observational studies (case–controls, cohort, and cross-sectional studies). The results of the quality assessment are presented in Supplementary Material Table S2.

## 3. Results

### 3.1. Study Selection

**Figure 1:**
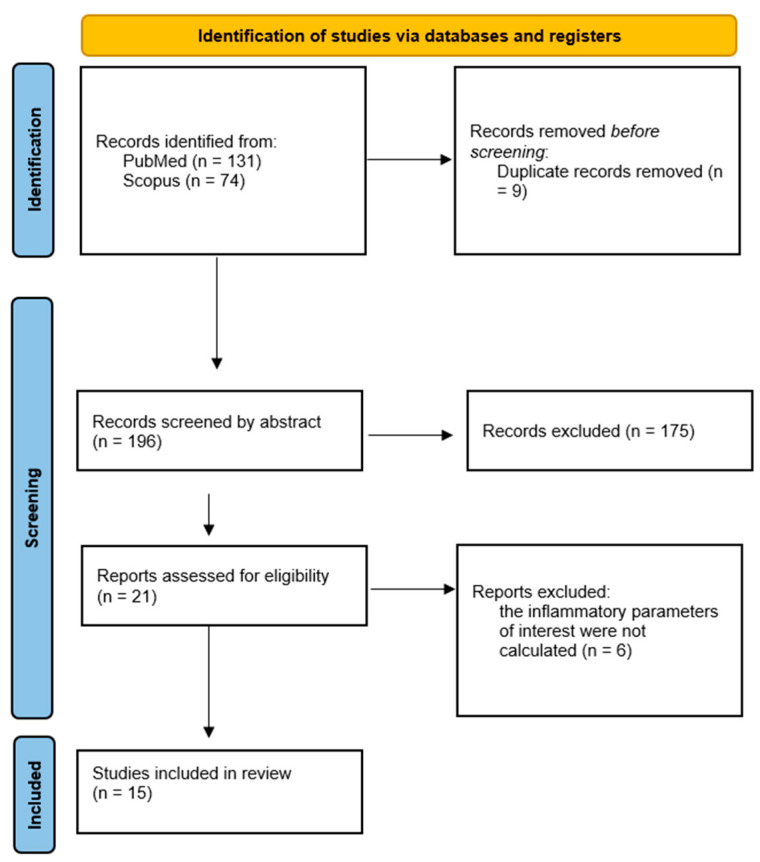
PRISMA flowchart [20].

Figure 1 illustrates the PRISMA flowchart. Initially, the search criteria generated 205 articles. After removing 9 duplicates, we excluded 173 articles after reviewing the titles and abstracts. Subsequently, based on the eligibility criteria, we identified 23 articles as potentially relevant to our review. After an exhaustive evaluation of the full texts, seven articles were excluded, leaving fifteen articles [21,22,23,24,25,26,27,28,29,30,31,32,33,34,35].

### 3.2. Characteristics of the Included Studies

We included 15 studies conducted in Turkey, China, Taiwan, the United States, and Israel, which evaluated the impact of methamphetamine, cocaine, cannabis (natural and synthetic), and opioid use on systemic inflammatory biomarkers.

Most studies (11/15) were retrospective case–control studies or cross-sectional in design, with recruitment in hospitals or outpatient consultations. Six studies investigated methamphetamine [21,22,23,24,25,26], one cocaine [27], four cannabis [28,29,30,31], and five opioids (one of them also studied cannabis) [31,32,33,34,35] abuse.

The sample size of the included studies ranged from 36 controls in the study by Turan Ç et al. [25] to more than 3700 participants in the population-based analysis by Alhassan HA et al. [30] The proportion of men was higher than 80% in most studies, with 100% male samples reported by Tanrıkulu et al. [22], Gürbüzer et al. [23], Turan et al. [25], Orum et al. [31], and Erdinc Cıcek et al. [32] Only a few studies included women, and in very scarce proportions, mainly in methamphetamine [21,24,26], cannabis [28,30], and opioid [33,34,35] groups, with no sex-stratified analyses performed. Biomarkers evaluated included NLR, PLR, MLR, hs-CRP, CAR, NAR, SII, BLR, ELR, MPV, and MHR, all obtained from routine blood counts and serum biochemical analyses (Table 3).

### 3.3. Risk of Study Bias

We evaluated the quality of the fifteen included studies according to the STROBE (Strengthening the Reporting of Observational Studies in Epidemiology) checklist, a 22-item tool designed to evaluate the quality of observational study reporting (Supplementary Data). The median score was 18/22 (range 15/22–21/22). The STROBE score for each study is shown in Table 3.

### 3.4. Summary of the Results

To facilitate the interpretation of the findings, the results were grouped according to the substance studied. Each section describes the characteristics of the included studies, the biomarkers evaluated, and the main findings of the study.

#### 3.4.1. Methamphetamine

We identified six studies that assessed the relationship between methamphetamine use and systemic inflammatory biomarkers.

Demir et al. [21] conducted a retrospective study in 84 patients with methamphetamine use disorder (MUD) and 81 controls, finding significantly lower NLR values (1.14 ± 0.58 vs. 1.52 ± 0.25; *p* < 0.001) and PLR (82.77 ± 22.6 vs. 10.5 ± 28.0; *p* < 0.001) in the MUD group. However, both indices were positively correlated with the daily consumption dose, suggesting a dose-dependent effect.

Tanrıkulu et al. [22] studied 139 men hospitalized for MUD and 139 controls, and no relation was found between MUD with NLR or PLR. The authors found significantly higher levels of CAR (0.083 ± 0.058 vs. 0.067 ± 0.031; *p* = 0.016) and NAR (0.108 ± 0.037 vs. 0.100 ± 0.031; *p* = 0.048) in the MUD group versus controls. In the multivariate analysis, CAR behaved as an independent predictor of the MUD group and was proposed as a useful inflammatory biomarker in this clinical context.

Gürbüzer et al. [23] conducted a prospective case–control study of 76 men with MUD and 70 matched controls, reporting significantly higher levels of NLR (2.24 ± 1.07 vs. 1.64 ± 0.43; *p* = 0.001), PLR (127.97 ± 39.10 vs. 108.62 ± 31.64; *p* = 0.004), MLR (0.29 ± 0.12 vs. 0.24 ± 0.07; *p* = 0.009), IBS (630.22 ± 301.53 vs. 423.12 ± 135.56; *p* < 0.001), CRP (7.21 ± 9.62 vs. 1.28 ± 1.19; *p* < 0.001), and MHR (0.02 ± 0.01 vs. 0.01 ± 0.004; *p* < 0.001) in the MUD group versus the control group. Daily methamphetamine intake was an independent predictor of IBS and was positively correlated with NLR, PLR, MLR, and neutrophils.

Ng et al. [24] included 122 patients with methamphetamine-induced psychosis (MIPD), 583 with schizophrenia, and 200 controls. Patients with MIPD presented significant increases compared to controls in NLR (2.74 ± 2.20 vs. 2.04 ± 0.94; *p* = 0.001), MLR (0.31 ± 0.18 vs. 0.24 ± 0.08; *p* = 0.013), and PLR (160.02± 8.71 vs. 149.47 ± 57.82; *p* = 0.005).

Turan et al. [25] included 50 patients with MUD and 36 controls, noting leukocytosis, neutrophilia, monocytosis, and thrombocytosis, along with significantly higher NLR (1.77 ± 0.37 vs. 1.52 ± 0.25; *p* = 0.045) and MLR (0.23 ± 0.05 vs. 0.16 ± 0.04; *p* < 0.001) in the MUD group compared with the control group. No differences were observed for PLR, and IL-6 levels were similar between groups.

Zang W et al. [26] (2023) included 632 patients with MUD and 325 controls. Patients with MUD had significantly higher NLR (3.84 ± 1.59 vs. 1.89 ± 0.54; *p*: < 0.001), PLR (149.79 ± 68.71 vs. 107.64 ± 35.61; *p* < 0.001), and MLR: (0.35 ± 0.19 vs. 0.18 ± 0.06; *p* < 0.001) compared with controls. In the multivariate analysis, NLR (OR = 71.7; *p* < 0.001) and MLR (OR = 6.3; *p* < 0.001) were confirmed as independent biomarkers of inflammatory status, while PLR lost significance. The AUC for NLR was 0.89 (sensitivity 76.7%, specificity 95.1%), and for MLR was 0.82 (sensitivity 72.6%, specificity 83.4%). NLR was positively correlated with severity score (SDS; r = 0.21; *p* < 0.001).

Taken together, (Table 4) the evidence suggests that methamphetamine use disorder (MUD) is associated with an increased systemic inflammatory profile, although findings are not completely homogeneous. While Demir et al. [21] reported lower NLR and PLR values with positive correlations to daily dose, most studies described significant increases in NLR, PLR, and MLR, as well as elevations in IBS, CRP, and monocyte/HDL ratio [23,25,26]. Tanrıkulu et al. [22] identified CAR as an independent predictor of MUD, proposing it as a clinical biomarker. Ng et al. [24] showed that patients with methamphetamine-induced psychosis have higher NLR, MLR, and PLR than controls. Zang et al. [26] further confirmed NLR and MLR as independent biomarkers of inflammation in MUD, with excellent discriminative capacity (AUC 0.89 and 0.82, respectively).

#### 3.4.2. Cocaine

We identified a single study that assessed the relationship between cocaine use and markers of systemic inflammation (Table 5). Soder et al. [27] conducted a case–control study in older adults (50–65 years), which included 107 individuals with cocaine use disorder (*CUD*) and 1309 age-matched controls from the NHANES cohort. NLR was measured from routine blood counts, and a propensity score-weighted regression model was applied to adjust for age, sex, race, socioeconomic status, and other substance use. Participants with cocaine use had significantly higher NLR compared to controls (2.38 ± 0.13 vs. 1.71 ± 0.02; *p* < 0.001), suggesting an increased inflammatory state associated with chronic cocaine use.

#### 3.4.3. Cannabis

We identified four studies that evaluated cannabis use and its relationship with inflammatory markers. Fridman et al. [28] conducted a retrospective cross-sectional study of 144 patients with schizophrenia, comparing 34 cannabis users with 110 non-users. No significant differences were found in NLR or MPV between groups (NLR: 2.00 ± 1.18 vs. 1.89 ± 0.82; not significant)

Guzel et al. [29] studied 40 synthetic cannabinoid (SG) users and 40 healthy controls (GCs), observing a significantly higher NLR in the consumer group (2.25 ± 0.99 vs. 1.81 ± 0.61; *p* = 0.019), with no differences in PLR

Alhassan et al. [30] analyzed data from 3715 cannabis users and 10,250 non-NHANES users (2005–2018) and found no differences in hs-CRP (3.5 vs. 3.7 mg/L; *p* = 0.65) or NLR (2.1 vs. 2.1; *p* = 0.89) after adjustment for sociodemographic and lifestyle factors.

Örüm and Kara [31] compared 56 males with marijuana use disorder (MUD), 56 with opioid use disorder (OUD), and 56 healthy controls. They found that marijuana users had higher monocytes (0.72 vs. 0.55 × 10^3^/μL; *p* = 0.018), higher monocyte percentages (7.96% vs. 6.87%; *p* = 0.010), and higher MLR (0.29 vs. 0.25; *p* = 0.049) than controls. No differences were observed in NLR (2.38 vs. 2.43; *p* = 0.97), PLR (103.49 vs. 107.90; *p* > 0.05), and MPV (7.91 vs. 7.52 fL; *p* = 0.665).

Overall, the available evidence (Table 6) indicates that natural cannabis use is not consistently associated with elevations in NLR or hs-CRP, both in the general population and in patients with schizophrenia [28,30]. However, data from Örüm and Kara [31] suggest that users with marijuana use disorder have higher monocyte counts and percentages, as well as higher MLR, which could reflect activation of the monocytic system, with no changes in NLR, PLR, or MPV. Only synthetic cannabinoid consumption is associated with significantly higher NLR [29].

#### 3.4.4. Opioids

We identified five studies (Table 7) that evaluated the association between opioid use (mainly heroin) and systemic inflammatory biomarkers.

Cıček et al. [32] conducted a prospective controlled study in 90 men with heroin dependence and 60 controls, finding significantly higher NLR values (1.86 ± 0.89 vs. 1.30 ± 0.31; *p* = 0.011) and PLR (112.0 ± 50.4 vs. 88.95 ± 24.6; *p* < 0.001) in OUD patients versus controls, with a positive correlation with disorder duration.

Guzel et al. [33] reported similar findings in 51 patients with heroin/opioid use versus 50 controls, showing elevated NLR and PLR (NLR: 2.31 ± 1.42 vs. 1.61 ± 0.95; *p* < 0.001; PLR: *p* = 0.014).

Baykara et al. [34] included 142 OUD patients and 140 controls, finding significantly higher NLR (2.32 ± 1.77 vs. 1.71 ± 0.79; *p* < 0.001) and MLR, but not in PLR, in the OUD group. The high prevalence of smoking in the OUD group (94.4% vs. 26.4% in controls) was a possible confounding factor.

In the study of Orum et al. [35] in 2018, they included 61 OUD and 61 controls. No significant differences in NLR (2.05 ± 1.23 vs. 2.22 ± 2.04; *p* = 0.808) or BLR (0.02 ± 0.01 vs. 0.03 ± 0.05; *p* = 0.099) were observed. However, the authors found significant differences for PLR (86.46 ± 27.10 vs. 101.76 ± 34.33; *p* = 0.012) and MLR (0.19 ± 0.06 vs. 0.25 ± 0.14; *p* = 0.005) in the OUD group versus the control group.

In a second study by Orum et al. [31] in 2020, including 56 OUD and 56 controls, only PLR was different between OUD patients and controls. (87.50 ± 25.46 (OUD) vs. 107.90 ± 39.80 (controls); *p* = 0.038)

Taken together, the available evidence suggests that opioid use disorder is consistently associated with elevated NLR and PLR, findings observed in both prospective and cross-sectional studies [32,33,34]. In addition, Baykara et al. [34] reported a significant increase in MLR.

## 4. Discussion

To our knowledge, this is the first review to analyze CBC-derived inflammatory biomarkers (NLR, MLR, PLR, etc.) between different unregulated substances abusers in an integrated manner (Table 8). The body of evidence suggests that methamphetamine and opioids are consistently associated with increased systemic inflammatory markers, especially NLR and MLR, which in several studies behave as independent predictors and are related to greater clinical severity [23,26,32,34]. For cocaine, although the evidence is limited to a single large cross-sectional study with propensity matching, higher NLR is also observed in chronic users [27]. For natural cannabis abuse, the findings are heterogeneous and, globally, do not show consistent elevations in NLR or hs-CRP. In contrast, synthetic cannabis tends to be associated with higher NLR, consistent with subclinical inflammation [28,29]. These results regarding cannabis abuse are consistent with the systematic review and meta-analysis by Moshfeghinia et al. [17], which found no global differences in NLR but did find alterations in other hematological parameters and IBS, with significant heterogeneity between studies [17].

The increases in NLR/MLR observed in methamphetamine are biologically plausible [4,7,36,37]. The substance activates microglia, promotes oxidative stress, and persistent transcriptional changes in the CNS, favoring a systemic proinflammatory tone (relative neutrophilia and relative lymphopenia) [4,7,36,37]. Preclinical studies show microglial activation, production of reactive oxygen species (ROSs), and release of cytokines such as IL-6 and TNF-α after exposure to psychostimulants [36,38]. In opioids, in addition to the classic immunomodulatory effects, interaction with TLR4 and activation of microglia, as well as MAPK and cGAS-STING signaling, have been demonstrated, contributing to neuroinflammation and a peripheral inflammatory state detectable in blood counts [15,18,19,39,40,41]. In cannabis, endocannabinoid signaling can modulate inflammation in opposite directions based on dose, chronicity, and cannabinoid mixture, which could explain the heterogeneity of clinical outcomes (no consistent changes in NLR, but variations in PLR, IBS, leukocytes, and monocytes in some studies) [17,28,42].

Systemic inflammatory markers values in substance abusers could be modified by associated lifestyle factors, including unstable housing or homelessness, poverty, incarceration, poor nutrition, unemployment, and concurrent mental health conditions. In fact, mental disorders [43] and poor nutrition [44] are strongly related to altered inflammatory markers. Furthermore, infections [45] are common in OUD due to poor living conditions, malnutrition, communal use of contaminated needles, and sexual intercourse with individuals with sexually transmitted diseases.

As described above, in 5 out of 15 studies only men were recruited [22,23,25,31,32]. Only a few studies included women in the population and in a very scarce proportions, mainly in the methamphetamine [21,24,26], cannabis [28], and opioid [33,34,35] groups. Only the large study by Alhassan HA et al. [30] included a majority of females (51.2%); however, no sex-stratified analysis of systemic inflammatory markers was performed. This male/female imbalance in the published studies significantly affects the generalizability of the findings.

The clinical relevance of evaluating systemic inflammatory markers in unregulated substance abusers would be considered regarding their predictive role in the following: (a) Disease related to substance abuse, including alcohol abuse disorder-related diseases such as hepatitis [46] or delirium tremens [47], as well as early diagnosis of depression [48] or severity of anorexia [49]. (b) Duration of MOP-r agonist intake, daily buprenorphine/naloxone dose, consumption route, severity of withdrawal symptoms, and level of self-reported pain. The inflammation group may require a higher daily dose of buprenorphine/naloxone due to their more severe withdrawal symptoms. Regression models demonstrated that Beck Depression Inventory scores and age are also significant predictors of inflammation [50]. (c) Poor clinical outcome in NSCLC patients treated with immune checkpoint inhibitors [51]. (d) Poorer response rates to biologically driven treatments in inflammatory bowel disease [52] or rheumatoid arthritis [53].

The neutrophil-to-lymphocyte ratio (NLR), which can be easily calculated from routine complete blood count tests, is recognized as a marker of systemic inflammation and immune system imbalance. Research has shown notable differences in NLR values between individuals diagnosed with substance use disorders and healthy control groups across various types of substances.

From a public health perspective, NLR’s affordability and widespread availability make it particularly useful in healthcare settings where care may be sporadic or fragmented, such as emergency rooms, community clinics, and correctional health services. Regular inclusion of NLR in clinical evaluations could help detect individuals at higher health risk earlier, facilitating timely preventive measures before serious health decline occurs. On a broader scale, analyzing population-level NLR data can assist in tracking inflammatory burdens and guiding the allocation of preventive resources in communities heavily affected by substance use.

Furthermore, as noted earlier, using biomarkers like NLR in clinical practice can enhance multidisciplinary care by providing an objective, shared metric that supports collaboration among primary care providers, addiction specialists, mental health professionals, and infectious disease experts. However, because NLR is not disease-specific, it must be interpreted carefully within relevant clinical and social contexts. Ethical application requires that its use be non-punitive and free from stigma, embedded within supportive care frameworks. In summary, NLR holds promise as a complementary tool in integrated public health and clinical approaches to improve outcomes for people with substance use disorders

Future directions include well-designed prospective studies to check the prognostic role of systemic inflammatory markers in the diagnosis of unregulated substance abuse disorder, disease severity, and their predictive role in the management of immune related diseases.

### Limitations

In the review of 15 included studies, the quality of reporting was variable. All but one of the complied studies fell between 15 and 20 of the 22 STROBE items, showing an adequate presentation of results and objectives, but with frequent deficiencies in areas such as confounders control, justification of sample size, and discussion of biases or external validity. Only one study achieved compliance with more than 90% (21/22) of the STROBE criteria [30], which shows the need to improve transparency and methodological rigor in observational research applied to substance use disorders.

The best scores were observed in studies that adjusted for age, sex, smoking, and comorbidities or employed propensity matching, although confounding factors such as smoking (very prevalent in OUD), subclinical infections, and psychotropic medication use persist. There was also heterogeneity in the definition of exposure (current vs. chronic use; natural vs. synthetic) and in the cut-off points for NLR and PLR, which explains some of the observed variability.

Another relevant finding is the marked sex imbalance: most studies included more than 80% men, and some included exclusively men, with very few analyses stratified by sex. This limits extrapolation of findings and underscores the need to include greater female representation and to conduct subgroup analyses that explore the role of sex hormones or gender factors in the inflammatory response.

Together, CBC-derived indices (NLR, MLR, PLR, IBS) are accessible, low-cost biomarkers that could help monitor inflammatory status in patients with substance use disorder. However, prospective multicenter studies are required, with standardization of methods, adjustment for confounding factors, and greater female representation, to clarify the temporal dynamics of inflammation (acute intoxication, withdrawal, maintenance treatment) and to evaluate the predictive value of these markers in clinical and psychiatric outcomes [40,41].

## 5. Conclusions

This review integrates, for the first time, the available evidence on inflammatory biomarkers derived from blood counts in users of different unregulated substances, showing that methamphetamine and opioid users consistently present an increase in NLR and MLR, with potential value as independent and accessible biomarkers to monitor inflammatory status [23,26,32,34,35,36,38,39]. For cocaine, although the evidence is limited to a single population-based study, a significant increase in NLR has been reported, supporting the hypothesis of a chronic proinflammatory state in users [27,40]. For natural cannabis, results are heterogeneous and show no consistent changes in NLR or hs-CRP, whereas synthetic cannabis is associated with higher NLR, which could reflect subclinical inflammation [17,28,29].

The integration of simple biomarkers such as NLR, PLR, MLR, and IBS into clinical practice could facilitate risk stratification and follow-up of patients with substance use disorder, given their low cost and wide availability. However, methodological heterogeneity, the predominance of males in samples, and the scarcity of prospective studies limit the generalizability of these findings. Longitudinal, multicenter studies with greater female representation are necessary to clarify the temporal course of inflammation and to evaluate its predictive value for clinical and psychiatric outcomes [39,40,41].

## Figures and Tables

**Table 1:** PECO question.

P (Population)	Adults (≥18 years), with unregulated substance use (cocaine, cannabis, opioids, MDMA, and methamphetamine), with or without a diagnosis of substance use disorder.
E (Exposure)	Use of such substances (current use or documented history).
C (Comparator)	Non-consuming individuals (healthy controls or general population without exposure).
O (Outcomes)	Markers of systemic inflammation: NLR, PLR, MLR, Hs-CRP, CAR, NAR, SII, BLR, ELR, MPV, and MHR.

**Table 2:** Markers of systemic inflammation.

NLR	Neutrophil/lymphocyte ratio
PLR	Platelet/lymphocyte ratio
MLR	Monocyte/lymphocyte ratio
Hs-CRP	Ultra-sensitive C-reactive protein
CAR	C-reactive protein/albumin ratio
NAR	Neutrophil/albumin ratio
SII	Systemic Inflammatory Index
BLR	Basophil/lymphocyte ratio
ELR	Eosinophil/lymphocyte ratio
MPV	Mean platelet volume
MHR	Monocyte/HDL ratio

**Table 3:** Characteristics of the included studies.

Author	Country	Design	Substance Exposure	Participants	Male/ Female	Inflammatory Indexes	STROBE
Demir B et al. 2021 [21]	Türkiye	Retrospective Case–Control	Methamphetamine	MG: 84 HG: 81	97.5% M 2.5% F	NLR PLR	17/22
Tanrıkulu AB et al. 2023 [22]	Türkiye	Retrospective Case–Control	Methamphetamine	MG: 139 HG: 139	100% M	NLR PLR MLR CAR NAR	18/22
Gürbüzer N et al. 2024 [23]	Türkiye	Prospective Case–Control	Methamphetamine	MUD: 76 Control: 70	100% M	NLR PLR MLR BLR YES MHR CRP (mg/L)	19/22
Ng MH et al. 2024 [24]	Taiwan	Retrospective Case–Control	Methamphetamine	MG: 122 SG: 583 HG: 200	88.5% M 11.5% F	NLR PLR MLR	20/22
Turan Ç et al. 2023 [25]	Türkiye	Retrospective Case–Control	Methamphetamine	MG: 50 HG: 36	100% M	NLR PLR MLR	18/22
Zang W et al. 2023 [26]	China	Prospective Case–Control	Methamphetamine	MG: 632 HG: 325	82,4%/17.6%	NLR PLR MLR	17/22
Soder HE et al. 2020 [27]	United States	Cross-sectional Case–Control	Cocaine	CUD: 107 age-matched controls 1309	86% M 14% F	NLR	19/22
Fridman J et al. 2023 [28]	Israel	Retrospective Cross-Sectional Study	Cannabis	144 schizophrenia patients: Cannabis users: 34 Non-users: 110	78% M 22% F	NLR MPV	15/22
Derya Guzel et al. 2017 [29]	Türkiye	Observational, Cross-Sectional with Control Group	Cannabis	SSG: 40 CG: 40	Unknown	NLR PLR	16/22
Alhassan HA et al. 2023 [30]	United States	Cross-Sectional Analysis of NHANES	Cannabis	Current users: 3715 Never users: 10,250	48.8% M 51.2% F	NLR hs-CRP	21/22
Orum MH et al. 2020 [31]	Türkiye	Retrospective Cohort Study with Controls	Cannabis Opioids	OUD: 56 MUD: 56 Controls: 56	100% M	NLR PLR MLR BLR	15/22
Erdin Cıček et al. 2018 [32]	Türkiye	Prospective Controlled Study	Opioids	PAT: 90 CG: 60	100% M	NLR PLR	17/22
Derya Guzel et al. 2018 [33]	Türkiye	Cross-Sectional Study with Control Group	Opioids	Heroin group: 51 GC: 50	96% M 4% F	NLR PLR	17/22
Baykara S et al. 2022 [34]	Türkiye	Prospective Cross-Sectional	Opioids	OUD: 142 CG: 140	98.6% M 1.4% F	NLR MLR PLR ELR BLR MPV	16/22
Orum MH et al. 2018 [36]	Türkiye	Retrospective Cohort Study with Controls	Opioids	OUD:61 CG:61	91.8% M 8.2% F	NLR MLR PLR BLR	15/22

MUD: methamphetamine use disorder; MG: methamphetamine group; SG: schizophrenia group; HG: healthy group; CUD: cocaine use disorder; SSG: study group with synthetic cannabis use; CG: control group; OUD: opioid use disorder; PAT: patients with heroin dependence.

**Table 4:** Results of methamphetamine studies.

Author	Defining Groups	Method Uses for Measurement	Main Findings (*p*-Value)	Limitations
Demir B et al. 2021 [21]	Patients diagnosed with DSM-5 methamphetamine use disorder at an addiction center (*n* = 84) compared with age- and sex-matched healthy controls (*n* = 81).	Fasting blood samples were obtained and automated blood count was performed in the central laboratory; NLR and PLR were calculated from neutrophil, lymphocyte, and platelet counts.	NLR < 0.001 PLR < 0.001	Small sample size, does not justify sample size, lacks multivariate analysis, no control of confounders, and only male sex.
Tanrıkulu AB et al. 2023 [22]	Patients diagnosed with methamphetamine use disorder based on DSM-5 criteria (*n* = 139) versus healthy controls (*n* = 139), all evaluated at the same teaching hospital.	Venous blood was collected on an empty stomach and processed in automatic analyzers for complete blood count and serum biochemistry; NLR, PLR, and MLR were calculated from cell counts, and CAR and NAR were obtained from ultra-sensitive PCR and serum albumin levels.	NLR = 0.574 PLR = 0.324 MLR = 0.388 CAR = 0.016 NAR = 0.048	It does not adjust for confusion due to comorbidities, lack of follow-up data, single-hospital sample, and without analysis of statistical power.
Gürbüzer N et al. 2024 [23]	Methamphetamine users with a confirmed diagnosis of methamphetamine use disorder (MUD) according to DSM-5 and structured clinical interview (*n* = 76) were compared with healthy controls (*n* = 70).	Peripheral blood samples were taken after 8 h of fasting and analyzed in automated systems for blood count and biochemical profiling; NLR, PLR, MLR, BLR, SII, and MHR were calculated from these data.	NLR = 0.001 PLR = 0.004 MLR = 0.009 BLR = 0.520 SII < 0.001 MHR < 0.001 CRP (mg/L) < 0.001	No sampling calculation, insufficient adjustment for confounding variable, selection for convenience, and lack of evaluation of prolonged exposure.
Ng MH et al. 2024 [24]	Three groups: patients with methamphetamine-induced psychosis (*n* = 122) diagnosed according to DSM-5, patients with schizophrenia (*n* = 583) according to clinical criteria and review of psychiatric history, and healthy controls (*n* = 200).	Complete blood count results obtained at hospital admission were used; NLR, PLR, and MLR were calculated retrospectively from absolute counts extracted from electronic clinical records.	NLR = 0.001 PLR = 0.22 MLR < 0.001	Without formal management of missing data, there is no adjustment for psychiatric treatments, possible diagnostic bias, and lack of socioeconomic discussion.
Turan Ç et al. 2023 [25]	Patients with methamphetamine use disorder confirmed by structured clinical interview (SCID-5) (*n* = 50) compared with a healthy control group (*n* = 36).	Fasting blood was drawn and processed by an automated hematology counter to obtain NLR, PLR, and MLR, and serum IL-6 and TNF-α levels were measured using commercial ELISA kits.	NLR = 0.045 PLR = 0.156 MLR < 0.001	Restricted sample, no losses or rejections, no multivariate adjustment, and omitted concomitant alcohol consumption/smoking as confounders.
Zang W et al. 2023 [26]	Prospective cohort of methamphetamine users diagnosed by DSM-5 and psychiatric clinical interview (*n* = 632) compared with healthy controls recruited from the same region (*n* = 325), matched by age and sex.	Blood samples were collected for complete blood count and routine biochemistry at a referral hospital, and NLR, PLR, and MLR were calculated following standardized protocols in an accredited laboratory.	NLR < 0.001 PLR < 0.001 MLR < 0.001	Selection of controls not very detailed, no assessment of socioeconomic status, lack of adjustment for lifestyle, and possible geographical bias.

MG: methamphetamine-induced psychotic disorder group; SG: schizophrenia group; HG: healthy group.

**Table 5:** Results of the cocaine studies.

Author	Defining Groups	Method Used for Measurement	Main Findings (*p*-Value)	Limitations
Soder HE et al. 2020 [27]	Adults diagnosed with cocaine use disorder (CUD) according to DSM-5 criteria. A total of 107 patients were included and compared with 1309 age-, sex-, and ethnicity-matched controls via propensity score matching.	Routine complete blood count; NLR calculated from absolute neutrophil and lymphocyte counts obtained from electronic medical records.	NLR < 0.001	Without multivariate analysis of confounding, single-institution setting, limitations with self-report of abuse, and without dose evaluation.

**Table 6:** Results of cannabis studies.

Author	Defining Groups	Method Used for Measurement	Main Findings (*p*-Value)	Limitations
Alhassan HA et al. 2023 [30]	Cannabis users identified by self-reported questionnaires in NHANES 2005–2018 (*n* = 3715) compared with non-users (*n* = 10,250).	Blood counts and hs-CRP obtained from NHANES; NLR was calculated from standardized cell counts.	hs-CRP *p* = 0.65 NLR *p* = 0.89	Without adjustment for psychiatric comorbidities, lack of control of nutritional factors, and no consumption pattern or medical histories are described.
Fridman J et al. 2023 [28]	Patients with schizophrenia diagnosed according to DSM-5; 34 cannabis users (confirmed by clinical interview and self-report) compared to 110 non-users.	Routine blood counts; NLR and MPV calculated retrospectively from electronic records.	NLR *p* > 0.05 (NS) MPV *p* > 0.05 (NS)	Unclear exclusion criteria, unadjusted confounders, lack of detail of statistical techniques, and does not evaluate chronic use of cannabis.
Guzel D et al. 2017 [29]	Synthetic cannabinoid users diagnosed by clinical interview and positive toxicology test result, compared with healthy controls (40 vs. 40).	Complete blood count processed in an automated analyzer; NLR and PLR calculation.	NLR *p* = 0.019 PLR *p* > 0.05	Only men, no sample size calculation, simple statistical analysis, and lack of adjustment for smoking and infectious diseases.
Orum MH et al. 2020 [31]	Patients with marijuana use disorder (MUD) diagnosed according to DSM-5 by structured interview (*n* = 56 per group) compared with 56 healthy controls.	Automated blood count; NLR, PLR, and MLR calculated from absolute blood cell counts.	MLR *p* = 0.049 PLR *p* > 0.05 NLR *p* = 0.970 BLR *p* = 0.403	A small sample, only males, absence of multivariate analysis, no reporting duration of consumption, and confounders such as tobacco are not included.

**Table 7:** Results of opioid studies.

Author	Defining Groups	Method Used for Measurement	Main Findings (*p*-Value)	Limitations
Guzel D et al. 2018 [33]	Patients with heroin use disorder diagnosed according to DSM-5 by structured clinical interview (*n* = 51) compared with healthy controls matched by age and sex (*n* = 50).	Complete blood count in automated analyzer; NLR and PLR calculated from absolute blood cell counts.	NLR *p* < 0.001 PLR *p* = 0.014	Limited to men, no adjustment for psychiatric comorbidities, insufficient control of previous inflammatory status, and lack of robust multivariate analysis.
Cıček E et al. 2018 [32]	Patients with heroin dependence diagnosed by DSM-5 and psychiatric interview (*n* = 90) compared with healthy controls (*n* = 60).	Routine blood count and biochemical analysis; NLR and PLR calculated from laboratory values.	NLR *p* = 0.011 PLR *p* < 0.001	Lack of sample calculation, no control for somatic comorbidities, simple confusion analysis, and no discussion of cultures or diets.
Baykara S et al. 2022 [34]	Patients with opioid use disorder (OUD) diagnosed according to DSM-5 and structured interview (*n* = 142) compared with healthy controls (*n* = 140).	Automated blood counts and biochemical parameters; NLR, MLR, PLR, ELR, BLR, and MPV calculation.	NLR *p* < 0.001 MLR *p* < 0.001 PLR *p* = 0.116 ELR *p* = 0.717 BLR *p* = 0.009 MPV *p* = 0.663	No adjustment for chronic clinical factors, limited to patients on treatment, samples from a single region, and lack of analysis by subgroups.
Orum MH et al. 2018 [35]	Patients with opioid use disorder (OUD) diagnosed according to DSM-5 by structured interview (*n* = 61 per group) compared with 561 healthy controls.	Automated blood count; NLR, PLR, and MLR calculated from absolute blood cell counts.	NLR *p* = 0.808 MLR *p* = 0.005 PLR *p* = 0.012 BLR *p* = 0.099	A small sample, 91% males, absence of multivariate analysis, no reporting duration of consumption, and confounders such as tobacco are not included.
Orum MH et al. 2020 [31]	Patients with opioid use disorder (OUD) diagnosed according to DSM-5 by structured interview (*n* = 56 per group) compared with 56 healthy controls.	Automated blood count; NLR, PLR, and MLR calculated from absolute blood cell counts.	NLR *p* = 0.970 MLR *p* > 0.05 PLR *p* = 0.038 BLR *p* = 0.403	A small sample, only males, absence of multivariate analysis, no reporting duration of consumption, and confounders such as tobacco are not included.

**Table 8:** Studies showing a significant relationship between most common inflammatory markers and unregulated substance abuse.

Substance	Total Number of Studies Included	NLR *	PLR *	MLR *	BLR *
Methamphetamine [21,22,23,24,25,26]	6	5/6	3/6	4/5	0/1
Cocaine [27]	1	1/1	----	----	----
Cannabis [28,29,30,31]	4	1/4	0/2	1/1	0/1
Opioids [31,32,33,34,35]	5	5/5	5/5	2/3	1/3

* proportions showed the number of statistically significant studies out of the number of studies analyzing the marker.

## Data Availability

No new data were created or analyzed in this study.

## Supplementary Materials

The following supporting information can be downloaded at: https://www.mdpi.com/article/10.3390/healthcare14020232/s1, Table S1: Detailed search strategies; Table S2: Summary of scores by study using the STROBE checklist.
