# Supplementary material for: Unregulated Substance Abuse and Systemic Inflammation Markers: A Review
Source: Healthcare (Basel). 2026 Jan 16;14(2):232. doi: 10.3390/healthcare14020232 (PMC12841071; doi:10.3390/healthcare14020232)
Supplement: Supplementary file 1 [file healthcare-14-00232-s001.zip › healthcare-4019721-supplementary.pdf]

Supplementary Material

TableS1. Detailed search strategies

Database	Search strategy
Pubmed<br>Scopus	"Cocaine and neutrophil to lymphocyte"
	"Cannabis and neutrophil to lymphocyte"
	"Opioids abuse and neutrophil to lymphocyte"
	"Heroin and neutrophil to lymphocyte"
	"Fentanyl and neutrophil to lymphocyte"
	"Morphine and neutrophil to lymphocyte"
	"Methadone and neutrophil to lymphocyte"
	"Buprenorphine and neutrophil to lymphocyte"
	"Methamphetamines and neutrophil to lymphocyte"

TableS2. Summary of scores by study using STROBE checklist.

(Author , year)	STROBE Compliance	Major limitations identified
**Demir et al., 2021 [21]**	17/22	It does not detail the selection process; there is a lack of description of biases; without justification of sample size; Little discussion about generalization.
**Tanrıkulu et al., 2023 [22]**	18/22	It does not explain the use of confounders; no information on data loss; incomplete description of exclusion criteria and temporal context.
**Gürbüzer et al., 2024 [23]**	19/22	Sample size calculation is lacking; statistical control for insufficient confounding; Representativeness or selection biases are not addressed.
**Ng et al., 2024 [24]**	20/22	No missing data management or sensitivity analysis is reported; minimal discussion of methodological limitations.

(Author , year)	STROBE Compliance	Major limitations identified
**Turan et al., 2023 [25]**	18/22	No information about loss or missing data; it does not dispute selection bias or generalization; without justification of the sample size.
**Zhang et al., 2022 [26]**	17/22	No confounders control reported; no control selection detail; it does not discuss methodological limitations or information biases.
**Soder et al., 2020 [27]**	19/22	Missing data handling and confusion adjustment are missing; the cross-sectional design limits causal inferences and is not sufficiently discussed.
**Fridman et al., 2023 [28]**	15/22	Unclear inclusion/exclusion criteria; no confusion control; without description of detailed statistical methods or methodological self-criticism.
**Guzel et al., 2017 [29]**	16/22	Incomplete exclusion criteria; no description of biases; without multivariate analysis or in-depth discussion of limitations.
**Alhassan et al., 2023 [30]**	21/22	High compliance ; there is a lack of discussion about generalization and possible residual confounding effects.
**Orum et al., 2020 [31]**	15/22	It does not justify sample size; no description of bias control; no multivariate analysis; limited methodological discussion.
**Cıcek et al., 2018 [32]**	17/22	It does not report the calculation of the sample size; no description of biases; without sensitivity analysis or discussion of external validity.
**Guzel et al., 2018 [33]**	17/22	There is a lack of information on the control of confounders and leaks; brief discussion without methodological self-criticism.
**Baykara et al., 2022 [34]**	16/22	It does not describe selection or control for bias; without the use of confounders; it does not report power analysis or discussion of external validity.

(Author , year)	STROBE Compliance	Major limitations identified
**Orum et al., 2018 [35]**	15/22	Lack of sample size calculation; Lack of control/adjustment for confounders; Lack of bias analysis; 95% confidence interval not reported; Missing participant flow diagram
